# Integrated molecular portrait of non-small cell lung cancers

**DOI:** 10.1186/1755-8794-6-53

**Published:** 2013-12-03

**Authors:** Vladimir Lazar, Chen Suo, Cedric Orear, Joost van den Oord, Zsofia Balogh, Justine Guegan, Bastien Job, Guillaume Meurice, Hugues Ripoche, Stefano Calza, Johanna Hasmats, Joakim Lundeberg, Ludovic Lacroix, Philippe Vielh, Fabienne Dufour, Janne Lehtiö, Rudolf Napieralski, Alexander Eggermont, Manfred Schmitt, Jacques Cadranel, Benjamin Besse, Philippe Girard, Fiona Blackhall, Pierre Validire, Jean-Charles Soria, Philippe Dessen, Johan Hansson, Yudi Pawitan

**Affiliations:** 1 Institut Gustave Roussy, Villejuif, France; 2 Department of Medical Epidemiology and Biostatistics, Karolinska Institutet, Stockholm, Sweden; 3 Faculty of Medicine, University of Leuven, Leuven, Belgium; 4 Royal Institute of Technology, Stockholm, Sweden; 5 Science of Life Laboratory, Karolinska Institutet, Stockholm, Sweden; 6 Technical University of Munich, Munich, Germany; 7 Tenon Hospital, Paris, France; 8 Institut Mutualiste Montsouris, Paris, France; 9 Manchester Cancer Research Centre, University of Manchester, Manchester, England; 10 Department of Oncology-Pathology, Karolinska Institutet, Stockholm, Sweden

**Keywords:** NSCLC, AC, SCC, LCC, Systems biology

## Abstract

**Background:**

Non-small cell lung cancer (NSCLC), a leading cause of cancer deaths, represents a heterogeneous group of neoplasms, mostly comprising squamous cell carcinoma (SCC), adenocarcinoma (AC) and large-cell carcinoma (LCC). The objectives of this study were to utilize integrated genomic data including copy-number alteration, mRNA, microRNA expression and candidate-gene full sequencing data to characterize the molecular distinctions between AC and SCC.

**Methods:**

Comparative genomic hybridization followed by mutational analysis, gene expression and miRNA microarray profiling were performed on 123 paired tumor and non-tumor tissue samples from patients with NSCLC.

**Results:**

At DNA, mRNA and miRNA levels we could identify molecular markers that discriminated significantly between the various histopathological entities of NSCLC. We identified 34 genomic clusters using aCGH data; several genes exhibited a different profile of aberrations between AC and SCC, including PIK3CA, SOX2, THPO, TP63, PDGFB genes. Gene expression profiling analysis identified SPP1, CTHRC1and GREM1 as potential biomarkers for early diagnosis of the cancer, and SPINK1 and BMP7 to distinguish between AC and SCC in small biopsies or in blood samples. Using integrated genomics approach we found in recurrently altered regions a list of three potential driver genes, MRPS22, NDRG1 and RNF7, which were consistently over-expressed in amplified regions, had wide-spread correlation with an average of ~800 genes throughout the genome and highly associated with histological types. Using a network enrichment analysis, the targets of these potential drivers were seen to be involved in DNA replication, cell cycle, mismatch repair, p53 signalling pathway and other lung cancer related signalling pathways, and many immunological pathways. Furthermore, we also identified one potential driver miRNA hsa-miR-944.

**Conclusions:**

Integrated molecular characterization of AC and SCC helped identify clinically relevant markers and potential drivers, which are recurrent and stable changes at DNA level that have functional implications at RNA level and have strong association with histological subtypes.

## Background

Lung cancer is one of the most prevalent and deadliest cancers; it accounts for 13% of all cancer cases and 18% of the deaths in 2008 [1]. Among European men, in 2011 lung cancer deaths are predicted to reach ~182,000, with a standardized rate of 37.6/100,000 person-years [2]. The rate among European women is still expanding and may reach 14 to 15 per 100,000 person-years in 2015 [3]. Despite recent advances in surgical and chemo/radiation therapies, the prognosis is very poor, with a 5-year overall survival rate of only ~15%, which has not improved over several decades.

The inability to cure lung cancer is related to the advanced stage of the disease at the time of diagnosis and the resistance of the disease to currently available anti-cancer drugs [4]. In its early stages lung cancer tends to be asymptomatic, so at the time of diagnosis most tumors are locally advanced (stage IIIB) or metastatic (IV). Furthermore, even among early-stage patients who are treated primarily by surgery with curative intent, 30-55% will develop and die of metastatic recurrence.

Human lung cancers are classified into two major subtypes, small cell lung cancer (SCLC) and non-small cell lung cancer (NSCLC), with the latter accounting for ~80% of all primary lung cancers. NSCLC represents a heterogeneous group of cancers, consisting mainly of squamous cell carcinoma (SCC) and adenocarcinoma (AC), and a much smaller fraction of large-cell carcinoma (LCC). Current clinical management and therapeutics depend on histopathological classification, which is reliable for surgical specimens, but often difficult to assess for small biopsies. This is a serious issue, because in ~70% of cases only small biopsies or cytology specimens are available [5].

The objectives of this study were to utilize integrated genomic data including copy-number alteration, mRNA, microRNA expression and candidate-gene full sequencing data to characterize the molecular basis of the distinctions between AC and SCC. Cancer genomes are often unstable and accumulate a large number of mutations and structural or copy-number changes. Thus we cannot tell which genomic changes are ‘drivers’ and which are ‘passengers.’ Availability of data from multiple sources has the potential to solve these problems, where, for example functional or physiological impacts of the DNA-level genomic changes can immediately be verified at RNA level. Integrated molecular characterization of AC and SCC may help identify clinically relevant potential drivers, which are recurrent and stable changes at DNA level that have functional implications at RNA level and have strong association with histological subtypes.

## Methods

### Patients and tissue samples

The present study was organized by the CHEMORES initiative (Chemotherapy resistance consortium), which is an EU funded (FP6) Integrated Project involving 19 academic centres, organizations for cancer research, and research-oriented biotechnology companies in 8 European countries.

Tissue samples from a cohort of 123 patients who underwent complete surgical resection at the Institut Mutualiste Montsouris (Paris, France) between 30 January 2002 and 26 June 2006 were analysed. Clinical characteristics are given in Table 1. The median age of patients was 63 years (range 41–85), 34 (28%) were female and 89 (72%) were male. The histopathology of all tumors was reviewed by the same pathologist (JvdO): 50 patients had SCC, 57 AC, 13 LCC and 3 unclassified. Using the new 7th edition TNM staging 56 were stage I, 25 stage II, 28 stage III and 4 stage IV. Adjuvant platinum based chemotherapy was administered to 61 patients. Fifty-nine patients experienced a relapse. Two-year relapse-free survival was 64%, and the median time to recurrence for the cohort was 5.2 years. After a median follow up of 40 months (range 0–92) 36 patients had died and 23 patients were alive with recurrence.

**Table 1:** Characteristics of the patients

	**n = 123 (100%)**
Age median (range)	63 (40.9-84.6)
Males n (%)	89 (72%)
Smoking Current	64 (52%)
Former	51 (42%)
Never	7 (6%)
Histology AC	57 (46%)
SCC	50 (41%)
LCC	13 (11%)
Other	3 (3%)
Stage 1	56 (50%)
2	25 (22%)
3	28 (25%)
4	4 (4%)
Adjuvant chemo (%)	61 (50%)

Characteristics of the patients in the study population.

This study was performed using snap-frozen tumor and adjacent normal lung tissue. Samples were handled according to the Tumor Analysis Best Practices Working Group [6]. Haematoxylin and eosin stained frozen sections, taken before and after the cutting of slides for analysis, revealed a median cell content of 85% (an inter-quartile range of 65% to 95%). All tissues were banked after written informed patient consent, and the study was approved by the Ethics Committee of Institut Gustave Roussy (IGR). Genomic investigations were performed at IGR, leader of the Genomic work-package of Chemores consortium, in the genomic center core facility certified ISO9001, labelled European reference and training center for Agilent technologies. Analyses were performed at IGR and Karolinska Institute, the leader of integrated analyzes work-package.

### Oligonucleotide aCGH

DNA samples were extracted from tissues using Qiagen QIAamp DNA Mini kit (Qiagen, Hilden, Germany). In each case, the normal tissue sample was used as the reference to its corresponding tumor sample. DNA was restriction digested and controlled by Agilent Bioanalyzer on DNA 7500 chips (Agilent Technologies, Santa Clara, CA, USA). The fragmented reference and test DNA were labelled with Cy3-dUTP or Cy5-dUTP, respectively, using Agilent Genomic DNA Labelling Kit PLUS. Samples were purified using Microcon YM-30 filters (Millipore, Billerica, MA). Hybridization was carried out on Agilent 244K arrays for 24 hours at 65°C in a rotating oven (Robbins Scientific, Mountain View, CA) at 20 rpm, followed by appropriate washing steps. Scanning was performed with an Agilent G2505C DNA Microarray scanner using default parameters. Quantification of Cy5 and Cy3 signals from scans was performed with Feature Extraction v10.5.1.1 (Agilent Technologies) using default parameters.

### aCGH data processing and analysis

Resulting raw signals and log2 (ratio) profiles were normalized and centered according to their dye composition (Cy5/Cy3) and local GC content. These profiles were segmented with the Circular Binary Segmentation algorithm [7] through its implementation in the DNAcopy package for R v2.8.1 using default parameters. DNA copy number imbalances were detected considering a minimum of 3 consecutive probes and a minimal absolute amplitude threshold that was specific for each profile, accordingly with its internal noise. This specific internal noise was computed as one-fourth of the median of the absolute log2 (ratio) distances across consecutive probes on the genome. Of the 128 aCGH hybridizations performed, 17 were discarded: 7 due to their clinical annotations, 2 due to anomalies in their normal reference, and 8 due to the bad quality of their profile, resulting in 111 usable profiles. All aCGH coordinates in this study are mapped against the human genome as defined by the UCSC build hg18.

To assess the discovery of the genomic regions with differential anomalies between the AC, LCC and SCC populations, ANOVA tests were performed on the segmented aCGH dataset. To account for multiple testing, p-values were transformed to false discovery rate (FDR) [8].

### Gene expression and microRNA microarray assay

The lysis of 40 to 50 frozen sections of 10 micron-thickness, cut from each NSCLC tissue sample was done using a Polytron homogenizer (Ultraturrax, IMLAB, Lille, France). The RNA extraction was performed with TRIzol® Reagent protocol (Invitrogen, Carlsbad, CA, USA). Total RNA was quantified and qualified with Nanodrop ND-1000 spectrometer and Bioanalyzer-2100 (Agilent Technologies).

For dual color Cy3 (normal samples) and Cy5 (tumor samples) labelling, Agilent Fluorescent Low Input Linear Amplification kit adapted for small amounts of total RNA (500 ng total RNA per reaction) was used, followed by purification of labelled probes by Qiagen RNeasy Mini kit and by a protocol provided by Agilent. Gene expression profiling was performed with dye-swap, using dual-color 244K Human exon array from Agilent (custom design with the content of the 44K Human genome plus 195,000 probes, one for each exon as defined in refGene list of UCSC build hg18 (http://genome.ucsc.edu/)). Hybridization was carried out for 17 hours at 65°C at 10 rpm, followed by washing steps. Scanned microarray images were analyzed by using Feature Extraction software version 10.5.1.1 (Agilent).

For the microRNA analysis, normal and tumor samples were hybridized on separate arrays. Agilent miRNA Microarray System with miRNA complete labelling and hybridization kit was used for Cy3 labelling. Briefly, isolated total RNAs were dephosphorylated, labelled with pCp-Cy3 and hybridized to Agilent 8x15K arrays for 20 hours at 55°C in a rotating oven (Robbins Scientific) at 20 rpm. Slides were washed and scanned for gene expression using an Agilent G2565C DNA microarray scanner using defaults parameters.

### Gene mutations analysis

Sequencing was performed at IGR and at the Royal Institute of Technology (Stockholm, Sweden). DNA was extracted with QIAamp DNA Mini Kit (Qiagen, Hilden, Germany). After PCR amplification of target exons, sequencing reactions were carried out using the BigDye® Terminator Cycle Sequencing Kit (Applied Biosystems, Forster City, CA). The primer sequences are available on request. Sequencing reactions were run on a 48-capillary 3730 DNA Analyzer®. Sequence analysis and alignment was performed with SeqScape® software (Applied Biosystems). All detected mutations were confirmed in at least one independent PCR reaction. In all 123 samples, full coding sequences of exons including oncogenic mutational hotspots were analyzed corresponding to: TP53 (NM_000546.4) exons 5–8; KRAS (NM_004448.2) exons 2 and 3; EGFR (NM_005228.3) exons 18–21; PIK3CA (NM_006218.2) exons 10 and 21; BRAF (NM_004333.4) exon 15; ERBB2 (NM_004448.2) exons 18, 20–24; KDR (NM_002253.1) exons 2, 26, 27 and 30; and AKT1 (NM_005163.2) exon 4.

### Gene-expression data processing and normalisation

All processing methods used for gene expression analysis were performed on the median signal from Agilent Feature Extraction raw data files using functions and packages collected in the R Bioconductor project [9] as well as custom written routines.

For gene expression data, dye-swap arrays were first combined (by taking the average of intensities) to obtained only one array per condition. This combination has the result of centering the M values (log2ratios) on zero. Then, flagged spots as well as control spots were removed. Normalization was then performed using the *normalizeWithinArrays* function from R package LIMMA [10].

For miRNA data, control spots were systematically removed, and flagged spots (gIsFeatNonUnifOL and gIsSaturated columns from raw files) were considered as missing values (“NA”). Array normalization was performed using the least-variant-set method [11].

### Differential expression analyses of miRNA expression

To assess differentially-expressed miRNA, we first estimated the fold changes and standard errors between two groups of samples by fitting a linear model for each probe with the lmFit function of LIMMA package in R. Then we applied an empirical Bayes smoothing to the standard errors from the linear model previously computed with eBayes function.

### Integrated genomics using Driver-Gene Search algorithm

**Figure 1:**
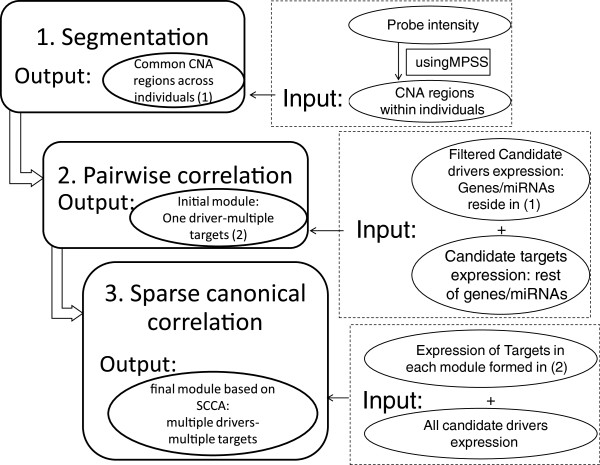
**Flowchart of DGS algorithm.** Driver and targets are identified in a three step process, as shown. Candidate drivers are firstly selected from genes/miRNAs that reside in copy-number altered (CNA) regions and filtered by various procedures, for example based on fold-change and consistency between expression level and copy number status. The rests of genes/miRNAs are candidate targets, which are grouped based on correlation with the candidate drivers. Correlation between all drivers and targets in each module is highlighted using sparse canonical correlation analysis (SCCA).

Motivated by Akavia *et al.*[12], we considered each gene in an altered region a potential driver; its functional impacts on other genes can then be due to its direct copy number alterations or indirectly via the corresponding expression of the gene. It was then assumed that the functional impact of the driver is mediated through a biological pathway, such as proliferation or invasion, which would contain genes that are correlated to the driver. We identified such pathways by clustering genes into the candidate drivers. Thus the output of this clustering process is a collection of potential drivers together with a set of target genes associated with each driver. We developed the Driver-Gene Search (DGS) algorithm for this purpose, employing transparent statistical analyses to identify the so-called candidate target and driver genes. A complete workflow is shown in Figure 1.

The DGS algorithm consists of 3 broad steps as follows. Step 1: Identify recurrent copy-number alterations (CNAs) across individuals. This is achieved using the combination of two computational algorithms implemented in MPSS and cnvpack, available from http://www.meb.ki.se/~yudpaw/. Based on a correlated random-effect model for the unobserved patterns, MPSS takes a robust smooth segmentation approach to identify whether a segment is a true CNA [13]. The segmentation threshold is fixed at −0.15 and 0.15 of the intensities for deletions and duplications, respectively. Segments with FDR less than 1e-05, number of probes less than 10, length of segments less than 1 kb are filtered out. As shown in Figure 1, these abnormal regions for individuals become input of step 1 in the workflow. We then apply the cnvpack to identify recurrent CNA regions [14], defined as alterations found in at least 10% of the subjects in whom at least one abnormal region is found by MPSS. Genes / miRNAs in recurrent CNAs are chosen as candidate drivers.

Step 2: Set up initial modules. First identify candidate drivers from CNA regions from Step 1, and compute pair-wise correlation between candidate driver and target genes. Each target gene is grouped with the highest significantly correlated candidate driver gene, using P < 0.001. From this initial pairing, we know that genes within each module are all highly correlated with at least one candidate driver. Step 3: Find final modules. It is reasonable to assume that these target genes are also possibly correlated with other candidate drivers. In this step, we identify the correlation between several candidate drivers and the target genes in every module using the canonical correlation analysis (CCA). We used an R package SCCA to perform sparse canonical correlation analysis [15].

### Network enrichment analysis

Next we functionally characterize the potential driver genes using the network enrichment analyses (NEA) [16]. Target genes are likely enriched with diverse biological processes and pathways that reveal the physiological roles of the driver and the associated genes. We use neaGUI to test whether there is an over-representation of network links between genes associated with their driver and genes in KEGG pathways. The tool is available for download from https://r-forge.r-project.org/projects/neagui/. To maintain an adequate number of genes for testing and a moderate correlation with the driver, we select a threshold of correlation coefficient based on Additional file 1: Figure S4 such that the number of target genes is around 100. For driver miRNA, the tested gene set contains biologically regulated genes obtained from miRBase [17]. The NEA method uses topological information of the gene interaction network, so target miRNAs identified by the DGS algorithm are not included in the NEA for testing.

### Validation of expression data analyses

For mRNA expression we used previously published NSCLC expression dataset GSE3141 [18] from the Gene Expression Omnibus website in order to validate some of the results based on mRNA expression. The dataset comprised of 58 AC and 53 SCC samples, and mRNA was extracted from frozen tissue of primary lung tumors. The gene expression was measured on Affymetrix Human Genome U133 Plus 2.0 Array, which we subsequently normalized using global normalization at the log-expression level. If a gene is represented by several Affymetrix probes, we take the average of the log-expression value to represent the gene. For miRNA expression we validated our results using Ming You *et al’*s data GSE29135 [19].

### Validation of candidate driver genes

For the candidate driver genes we also attempted to verify the CNA status and the correlation of CNA and expression in an independent dataset previously published by Chitale *et al.*[20]. Frequency of CNAs was validated in 199 AC cases hybridized to Agilent 44K CGH arrays, where probes were less dense than the 244K arrays used in our sudy. Of the 199 samples, 102 were hybridized to the HG-U133A 2.0 Affymetrix oligonucleotide arrays. The robust multichip average method, which outputs log2-transformed expression values, was used to summarize probe measurements into one measurement for each probe. We assessed copy number status of the 500 kb region surrounding each driver gene. On average, 20 probes were targeted at the driver gene region. A region was considered as amplified if its regional averaged probe intensity was significantly higher than zero, which was the global mean after standardization of the LogRatio intensities within samples. One-sided Welch t-test was used to assess the statistical significance.

## Results

### Copy-number alteration profiles

**Figure 2:**
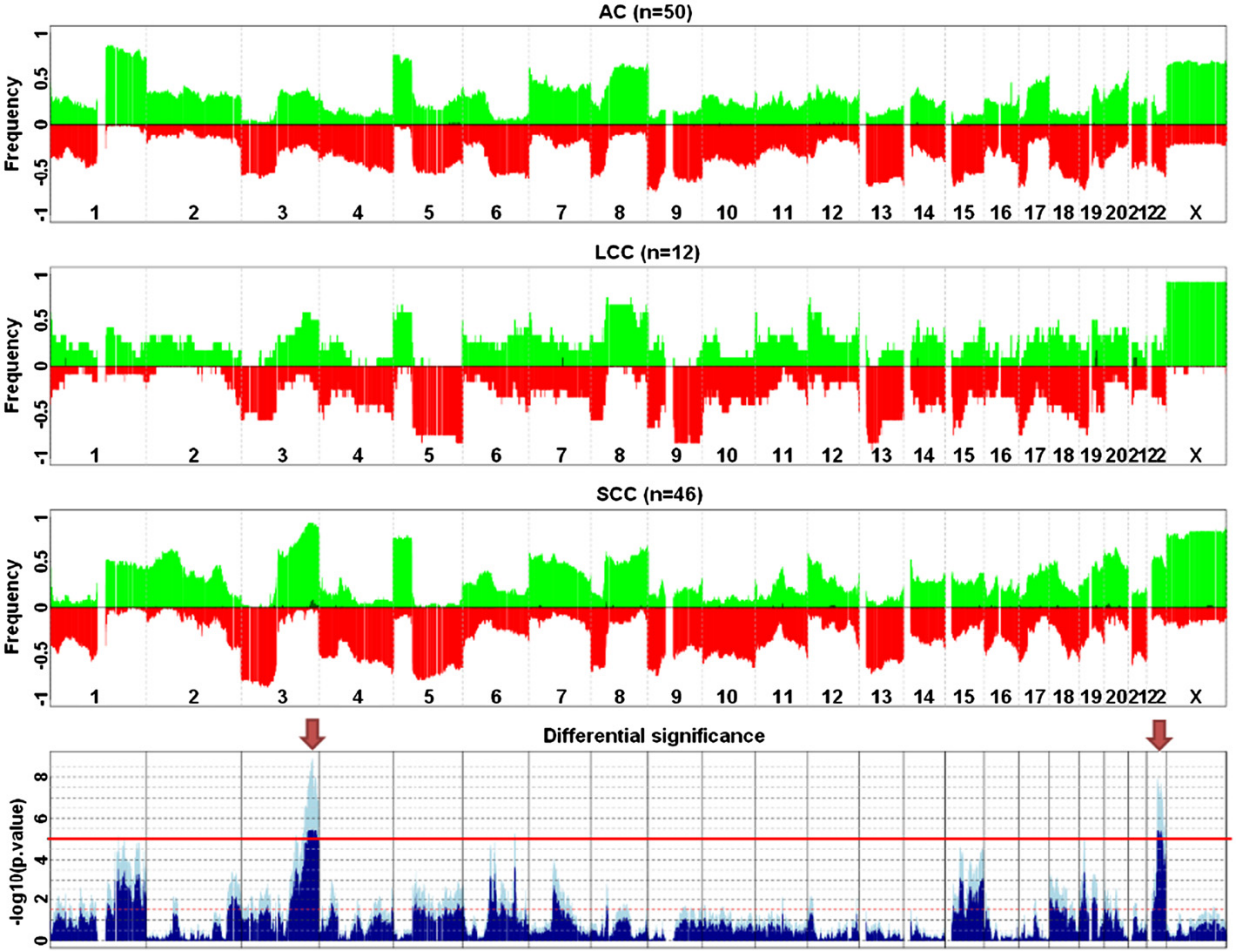
**Differential genomic regions for AC vs LCC vs SCC populations aCGH profiles.** The three upper panels display the average profiles of AC, LCC and SCC subpopulations as their respective frequencies of gains (green, from 0 to 100%) and losses (red, from 0 to −100%) along the human genome. Darker green bars correspond to the frequencies of amplifications, defined as regions with a log2 (ratio) above 1.0. The lowest panel shows the significance of the ANOVA tests displaying the minus log10-transformed raw (lighter blue) and BH-adjusted (darker blue) p-values. The horizontal red line corresponds to a BH-adjusted p-value < 1.0E-05. Arrows point to the two most significant differential regions: 3q26.2-3q29 and 22q12.1-22q13.1.

ANOVA testing was performed to compare the different histological groups according to their genomic anomalies from the aCGH data. At FDR < 1.0e-5, we identified 168 differential genomic regions. These regions were then merged into 34 genomic clusters within which the frequencies of aberrations for each genomic state and each subpopulation did not vary for more than 1%. These 34 clusters distributed into two main loci: chromosomal regions 3q26.2-29 and 22q12.1-13.1 (Figure 2). Additional file 1: Table S1 shows the top 34 differential genomic clusters and their genomic annotation of interest, and the gene composition is given in Additional file 1: Table S2.

### mRNA expression analyses

**Figure 3:**
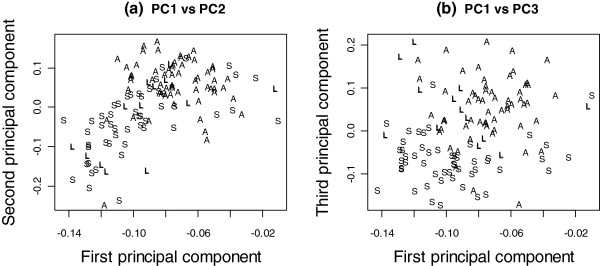
**Principal component plots of the mRNA data.** The first and second principal component plot (left) and the first and third principal component plot (right) of the mRNA data revealed the separation of squamous-cell carcinoma (S) from the adeno-carcinoma (A) and large-cell carcinoma (L).

Figure 3 shows the plots of the first 3 principal components (PC) of mRNA expression data. These plots indicate that the transcriptomic variability in NSCLC is dominated by histological types. Particularly, we observed in Figure 3(a) that AC was well separated from SCC. Indeed, a 15-gene classifier between AC and SCC achieved a cross-validated AUC of 96%, which means that these two types could almost perfectly be separated. The classifier was built using L1-penalized logistic regression, such that the estimates of many regression coefficients are shrunk towards zero. The selected 15 genes have the strongest effect with little shrinkage and can be used to predict AC and SCC. The clear separation between AC and SCC is also shown by the color map in Additional file 1: Figure S1. We validated this result using Bild *et al*’s NSCLC data GSE3141 [18], where the 15-gene signature achieved an AUC of 92%. We performed a Monte-Carlo test by taking 1000 random selections of 15 genes and considered each as a signature. For these random signatures we obtained a median AUC ~73%, and all had AUCs < 92%, thus giving a p-value < 0.001 for the observed signature. The list of the 15 classifier genes and the probes from both platforms are given in Additional file 1: Table S3.

In Figure 3(b) it appears that LCC and SCC form separate groups. LCC was only a small (n = 12/123) subset of non-small-cell lung cancers; interestingly, while its mRNA profile was not easily distinguished from that in AC (AUC < 50%), it was well separated from SCC (AUC =91%).

The gene expression profiling analysis identified a panel of 5 secreted biomarker candidates SPP1, CTHRC1, GREM1, SPINK1 and BMP7, and 5 non-secreted genes KRT6A, TP63, LGALS7, GCNT3, SPRR2D as IHC biomarker candidates (Table 2). The distributions of the markers are shown in Additional file 1: Figures S2 and S3. As shown in these figures, the corresponding distributions of these markers in Bild *et al*’s NSCLC data GSE3141 [18] were largely similar to those in our data. The list of the genes and the probes from both platforms are given in Additional file 1: Table S4. SPP1, CTHRC1 and GREM1 are candidate biomarkers to identify the cancer. Additionally, based on the AUC values in Table 2, SPINK1 and BMP7 would help to distinguish AC from SCC. The non-secreted IHC biomarkers can be used for annotation of small biopsies in order to identify the cancer and, except for GCNT3, they are informative for distinguishing AC from SCC.

**Table 2:** List of top 5 secreted and 5 non-secreted markers

**Candidate**	**Gene name**	**Frequency of overexpressionT vs N in AC patients (%) (p-value)**	**Frequency of overexpression T vs N in SCC patients (%) (p-value)**	**Chemores AUC (p-value)**	**Bild *et al. *** **AUC (p-value)**
SPP1	Secreted phosphoprotein 1	96	98	0.41	0.40
		(<10^-10^)	(<10^-10^)	(0.70)	(0.76)
CTHRC1	Collagen triple helix containing 1	96	98	0.42	0.40
		(<10^-10^)	(<10^-10^)	(0.63)	(0.75)
GREM1	Gremlin 1	88	98	0.40	0.40
		(<10^-10^)	(<10^-10^)	(0.77)	(0.78)
SPINK1	Serine peptidase inhibitor Kazal type 1	93	80	0.80	0.81
		(<10^-10^)	(1.14 × 10^-7^)	(0.03)	(0.01)
BMP7	Bone morphogenetic protein 7	34	90	0.86	0.82
		(0.01)	(<10^-10^)	(0.003)	(0.01)
KRT6A	Keratin 6A	73	96	0.90	0.88
		(9.18 × 10^-5^)	(<10^-10^)	(0.002)	(0.002)
TP63	Tumor protein p63	39	98	0.95	0.85
		(0.09)	(<10^-10^)	(<0.001)	(0.01)
LGALS7	Lectin, galactoside-binding, soluble, 7	73	94	0.87	--
		(9.18 × 10^-5^)	(<10^-10^)	(0.003)	--
GCNT3	Glucosaminyl (N-acetyl) transferase 3	88	80	0.72	0.68
		(<10^-10^)	(1.14 × 10^-7^)	(0.08)	(0.10)
SPRR2D	Small proline-rich protein 2D	52	96	0.84	--
		(0.76)	(<10^-10^)	(0.003)	--

Notes: T = tumour, N = Normal; Overexpression is defined as log(T/N) > 0. P-values in the brackets are from the test that whether the frequency differs from 50%. AUC values refer to prediction of AC vs SCC. Median values of individual AUCs of the ten biomarkers in classifying histology type are computed. Monte-Carlo p-values based on 1,000 runs are indicated in the bracket. LGALS7 and SPRR2D probes were not in Bild *et al.* data.

List of top 5 secreted (the first five entries) and 5 non-secreted markers (the second five entries) that are over-expressed in tumours compared to normal tissues.

### Driver genes analyses

In total we identified 864 CNAs occurring at least once among 86 subjects, where 55 CNAs occurred in at least 12 (10%) of the total 121 subjects. These 55 highly recurrent CNAs covered ~50% of the genes contained in the original 864 CNAs. To further increase our confidence we excluded regions that contained both amplifications and deletions: this resulted in 7 CNAs containing only amplifications and 6 CNAs with only deletions. These CNA regions contained 93 genes. The corresponding genes were further filtered out if their mRNA expression did not vary across subjects (i.e., standard deviation < 0.25); this step resulted in 75 genes in amplified regions and 7 genes in deleted regions.

To explore the impact of CNAs on gene expression, we analyzed the patterns of gene expression in samples with mutations compared to samples with normal copy number. We first note that many amplified genes do not necessarily have a higher expression in tumor compared to in normal tissue and vice versa, suggesting that further filtering is necessary. We thus kept only those genes that (1) exhibited significantly up-regulated gene expression in samples with amplifications compared to non-mutated samples, based on P < 0.05 using one-sided Welch’s t test and fold-change > 2; vice versa for samples with deletions, and (2) showed consistent over-expression in tumors with amplifications; vice versa in tumors with deletions. Using these requirements, all genes with recurrent copy number losses were excluded, and 8 genes with recurrent copy number gains were identified as a final list of candidate driver genes: RNF7, NDRG1, FAM49B, MRPS22, SLC25A36, ACPL2, PPP1R16A and LRRC14.

An initial pairing of candidate target and driver genes based on correlation formed 8 modules consisting of 8,600 target genes. The biggest module contained 2,147 target genes and smallest module 336 genes. Using SCCA, we reduced the number of target genes in the modules to an average of 815 target genes. The number of target genes in a module gives us some idea of the importance of a driver gene. It is tempting to speculate that the higher number of targets whose expression is affected by a driver the more likely the mutated driver in this module might play a major role in tumor development. We plotted the number of target genes as a function of the correlation threshold in Additional file 1: Figure S4. The ranking of the candidate driver genes remained the same across the correlation threshold.

We then compared the mRNA expression in samples with vs those without altered genes, and selected four candidate driver genes with fold-change > 4 and P < 0.001: MRPS22, RNF7, NDRG1, FAM49B; see Additional file 1: Figure S5. Next we assessed their predictive value in predicting tumor histology. The individual AUCs using of these four genes were 0.81 (Pvalue = 0.02), 0.75 (0.05), 0.73 (0.07), 0.40 (0.76), respectively; see Table 3. The Pvalues were obtained from a Monte-Carlo test by computing the AUCs of 1000 random genes. We validated this result using Bild *et al’*s data GSE3141, where the candidate driver genes achieved AUCs of 0.74 (Pvalue = 0.04), 0.81 (0.01), 0.72 (0.05), 0.40 (0.77). Thus MRPS22, RNF7 and NDRG1 were significant predictors of histology, but FAM49B does not appear to be specific to any histological type. MRPS22 was over-expressed in 78% and amplified in 16% of the SCC tumors, but showed little amplification or over-expression in AC (Additional file 1: Table S5). RNF7 and NDRG1 showed more amplification and mRNA over-expression in SCC compared to AC.

**Table 3:** AUCs of the candidate driver genes and miRNAs

	**Rank 1**	**Rank 2**	**Rank 3**	**Rank 4**
Genes	MRPS22	RNF7	NDRG1	FAM49B
Chemores	0.81 (0.02)	0.75 (0.05)	0.73 (0.07)	0.40 (0.76)
Bild *et al.*	0.74 (0.04)	0.81 (0.01)	0.72 (0.05)	0.40 (0.77)
miRNAs	hsa-miR-944	hsa-miR-570	hsa-miR-16-2*	hsa-miR-31*
Chemores	0.88 (0.001)	0.59 (0.10)	0.56 (0.18)	0.40 (0.61)
Ming You *et al.*	0.78 (<0.001)	0.52 (0.44)	0.44 (0.76)	0.56 (0.31)

Median values of individual AUCs of the four candidate driver genes and the four candidate driver miRNAs in classifying histology type. Monte-Carlo p-values based on 1,000 runs are indicated in the bracket.

Next we used an independent data set published by Chitale *et al*’s data [20] to get a bioinformatics validation of the potential driver genes. To assess the copy-number alteration profiles, Chitale *et al.* used Agilent 44K CGH arrays, which are much less dense than the 244K arrays in our study. Because the sensitivity of CNV detection algorithms is limited by the resolution of the array, we decided to validate the frequency of copy number gains for the candidate driver genes directly, as well as their properties, including the number of correlated genes and relationship between the copy number status and gene expression. (See more details in the Methods Section.) We did find significant copy-number gains for these driver genes. Using a threshold of p-value < 0.001, the frequency of copy number gains was 11.6%, 28.1% and 7.5% for MRPS22, NDRG1 and RNF7, similar or exceeding as we reported in Additional file 1: Table S5 for AC patients from our study. We then performed a one-sided Welch t-test to compare the gene expression level in patients with copy number gains vs the non-mutated samples. We obtained p-values of 0.07, 7.5 × 10^-6^, and 0.2 for MRPS22, NDRG1 and RNF7, respectively. Had we used the p-value threshold of 0.05 in defining the copy number gain, all three candidate driver genes would exhibit significantly up-regulated gene expression in samples with amplifications, with corresponding P-values 0.002, 6.7 × 10^-7^, and 0.0009, respectively, suggesting that expression of the three drivers exhibit the expected positive correlation between the copy number gains and up-regulated gene expression. We also find that the number of genes correlated with driver gene MRPS22, NDRG1 and RNF7 is 395, 219 and 311, respectively, at a correlation coefficient at least 0.4. This large number of correlated genes is similar with what we observe in our data in Additional file 1: Figure S4.

### Driver miRNAs

We used the same procedure to identify potential driver miRNAs and examined their predictive ability on histology type. Because of the total number of miRNAs was much smaller than the number of genes in the genome, less stringent filtering procedures were applied. We started with the 864 common CNAs. To further increase our confidence we excluded regions that were amplified in some individuals and deleted in others, but by allowing a mixture of amplifications and deletions at a threshold of less than 10% mixture, we arrived at 84 CNAs containing mainly amplifications and 254 CNAs with mainly deletions. These CNA regions contained 33 miRNAs. Expression of all these altered miRNAs has a standard deviation > 0.25.

To explore the impact of CNAs on miRNA expression, we analyzed the patterns of miRNA expression in samples with mutations compared to samples with normal copy number. We kept only those miRNAs that (1) exhibited up-regulated expression in samples with amplifications compared to non-mutated samples, based on P < 0.01 using one-sided Welch t test; vice versa for samples with deletions, and (2) showed over-expression on average in tumors with amplifications, and under-expression in tumors with deletions. Using these requirements, three miRNAs hsa-miR-16-2*, hsa-miR-570 and hsa-miR-944 with recurrent copy number gains were found, and one miRNA hsa-miR-31* with recurrent copy number losses was identified as a final list of candidate driver miRNAs.

Next we assessed the predictive ability of the four potential driver miRNAs in classifying tumor histology; Table 3. Only hsa-miR-944 showed a significant AUC of 88% (Pvalue = 0.001 from the Monte-Carlo test). We validated this result using Ming You *et al’*s data GSE29135 [19], where hsa-miR-944 achieved a median AUC of 78% (Pvalue = 0.001). None of the other miRNAs was significant on the validation data.

### Network enrichment analyses

The network enrichment analysis was used to functionally characterize the candidate driver genes and miRNA. In this analysis we assessed the gene-network interaction between the target genes of each candidate driver with the genes in KEGG pathways; this was previously shown to be more powerful than the gene-set enrichment analysis [16]. For each of the three potential driver genes that were also good predictors of histological subtypes, we used the number of target genes in Additional file 1: Figure S4 to guide the choice of threshold of correlation coefficient. A total of 72 target genes of MRPS22 were selected at a correlation threshold 0.6, and 68 and 113 target genes of NDRG1 and RNF7, respectively, at a correlation threshold 0.45. A set of 641 genes was regulated by hsa-miR-944, which best classifies tumor histology among the potential driver miRNAs. The expressions of a miRNA and its target mRNAs are not necessarily correlated, so we did not filter the set of target mRNAs of hsa-miR-944 by correlation coefficient.

**Figure 4:**
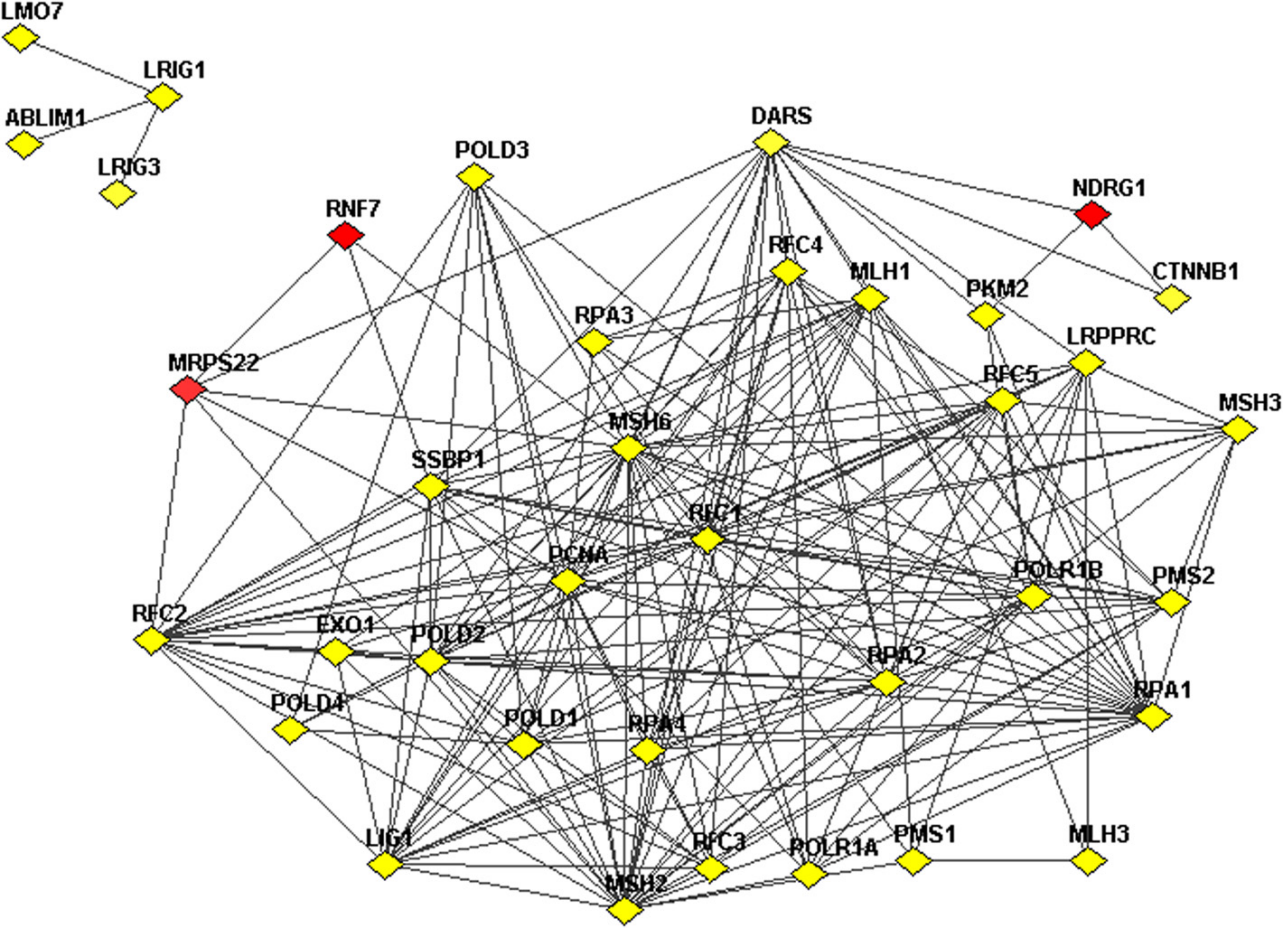
**Network links between 23 genes in pathway of mismatch repair and three driver genes.** Network links between 23 genes in pathway of mismatch repair and driver genes MRPS22, NDRG1, RNF7. Links shown include physical interactions, metabolic and signaling links from the functional coupling network (http://FunCoup.sbc.su.se).

The results are presented in Additional file 1: Table S6. For MRPS22 and hsa-miR-944, the top enriched pathways included many pathways known to be involved in cancer, such as DNA replication, cell cycle, mismatch repair, p53 signalling pathway and other lung cancer related signalling pathways. Intriguingly, the top pathways associated with NDRG1 included few known cancer-related pathways, but a large number of immunological pathways, such as Fc gamma R-mediated phagocytosis, B-cell receptor signalling pathway, T cell receptor signaling pathway, Fc epsilon RI signaling pathway and chemokine signaling pathway. RNF7 appeared to be associated with cell shape and motility-related pathways, such as focal adhesion, ECM-receptor interaction and regulation of actin cytoskeleton and adherens junction pathways. Figure 4 shows an example of network from the three driver genes and 23 genes in the pathway of mismatch repair, which was significantly enriched in the target genes of MRPS22. We observed more links connecting to MRPS22 than to RNF7 and NDRG1, in line with the results that the mismatch repair pathway was among the top enriched pathways for MRPS22, but not for the latter two drivers.

### Mutation analyses

We observed the following number of mutations from the sequence analyses: KDR 5, AKT1 0, KRAS 20, EGFR 13, PIK3CA 2, BRAF 4, ERBB2 1 and TP53 29 mutations. Further analyses were performed with the KRAS, EFGR and TP53 mutations. There was no evidence of association with time to relapse and no evidence of interaction with adjuvant chemo therapy (smallest P > 0.21 for the three and combined mutations). There was no significant association with tumor stage or lymph-node status.

The most significant result was the association with histology: almost all (18/20) KRAS mutations were found in AC, and none in SCC; 19% of AC had TP53 mutations vs 28% of SCC, but this was not statistically significant (P = 0.41). Since AC and SCC had significantly distinct mRNA expression profiles, we also found significantly distinct mRNA profiles between KRAS + vs KRAS- tumors (AUC = 0.86). For TP53 mutations, there were numerous DE genes (the top 200 genes had FDR ~ 8%), but the AUC for the L1 classifier was only ~0.67. This suggests that the multi-functionality of TP53 and its aberrations led to a broad spectrum of transcriptional responses.

## Discussion

In this study we analyzed a cohort of corresponding tumor and non-tumor tissue samples at DNA, RNA and miRNA level from NSCLC patients. Integrated molecular characterization of AC and SCC had identified clinically relevant markers and potential drivers, which are recurrent and stable changes at DNA level that have functional implications at RNA level and have strong association with histological subtypes.

The common feature of all of the previous studies was the exploration of tumor samples only. In contrast, the strength of our study design is the use of paired tumor and adjacent normal lung tissue from the same patient. This investigation was performed, for each patient, by comparing perfectly defined histological tumor and normal frozen lung samples obtained during curative surgery. The main advantage of this unique feature is that noise related to the genetic background variability is reduced, which should lead to more tumor-specific molecular data and more sensitive statistical analysis.

From a methodological perspective, our study is the few recent studies [21-24] to employ an integrative systems biology approach in NSCLC. We found distinct molecular characteristics of AC and SCC, a result supported also by others [25,26]. We also have evidence that LCC does not form a distinct molecular subgroup, but appears to be a subgroup of AC. Among the 34 genomic clusters identified by aCGH, several genes exhibited a different profile of aberrations between AC and SCC, including PIK3CA, SOX2, THPO, TP63, PDGFB genes. These chromosomal regions could be targeted by FISH probes, which might help pathologists to distinguish between the two entities. Thus far the FISH method was mainly used on the one hand to check for certain chemotherapy targets such as EGFR [27] or HER-2 [28]; and on the other hand to define a set of four genetic markers (frequent copy number gains in chromosomes 1q32, 3q26, 5p15, and 8q24) applied to spiral CT-guided FNA cytology samples, which were highly sensitive for the diagnosis of lung cancer and highly specific in their ability to exclude cancer within a given specimen [29].

Our results provide evidence that there are multiple potential biomarkers for early diagnosis and to help pathologists to distinguish between AC and SCC in small biopsies or in blood samples. Besides confirming some existing non-secreted SCC markers such as TP63 and KRT6A, novel findings include using gene expression profiling analysis to identify five secreted genes, among which SPP1, CTHRC1 and GREM1 would be candidate biomarkers to identify the cancer using blood samples; additionally, SPINK1 and BMP7 would help to distinguish AC from SCC as complementary to existing SCC markers. High expression of SPP1, also known as osteopontin, was associated with poor survival of patients with stage I NSCLC [30]. CTHRC1 is known to have an aberrant expression in many tumor types, such as cancers of the gastrointestinal tract, breast, lung and thyroid, but until now this marker was not investigated in differential diagnostics of NSCLC [31]. GREM1 is a member of the aberrantly activated Hedgehog signalling pathway, and has been reported to act in an oncogenic manner in lung adenocarcinoma and can induce cell migration, invasion and proliferation [32,33]. SPINK1 or tumor-associated trypsin inhibitor (TATI) did not appear to be a good tumor marker in lung cancer, since its sensitivity was poor and the correlation between TATI serum levels and stage of the disease and histological type was weak [34]. A recent publication indicated that BMP7 plays a key role in the regulation of lung cancer progression, linking its expression level to lymph node involvement [35].

Using integrated genomics approach we found in recurrently altered regions a list of three potential driver genes, MRPS22, NDRG1 and RNF7, which were consistently over-expressed in amplified regions, had wide-spread correlation with an average of ~800 genes throughout the genome and highly associated with histological types. Using a network enrichment analysis, the targets of these potential drivers were seen to be involved in DNA replication, cell cycle, mismatch repair, p53 signalling pathway and other lung cancer related signalling pathways, and many immunological pathways. NDRG1 has been recently reported to predict tumor angiogenesis and poor outcome in patients with lung cancer [36]. Expression of RNF7 has been found to be a prognostic marker in non-small cell lung cancer [37].

Multiple sets of molecular signatures are presented in this study. They are identified in parallel using different approaches with different intentions and could help to better characterize NSCLC from various aspects. Ten markers, composed of a panel of 5 secreted and 5 non-secreted biomarker candidates, are identified as top-ranking differentially expressed genes, i.e. by comparing the direction of relative mRNA expression between tumour vs normal tissues. In clinical practice, these potential biomarkers may be used for early diagnosis of the cancer, and additionally to distinguish between AC and SCC in small biopsies or in blood samples. In contrast, three potential drivers are selected using an integrated genomics approach, called Driver-Gene Search algorithm, which combines information on both of DNA and RNA levels. The DGS algorithm attempts to select potential driver genes that may have functional impact on the expression of other genes due to copy-number alterations of the driver genes. There is no guarantee that the driver genes are ranked as the top differentially expressed genes. The findings of the drivers provide a clue for downstream experimental analysis to understand the molecular mechanisms of the development of lung cancer.

## Conclusions

In summary, an understanding of the molecular mechanisms involved in formation of various NSCLC subtypes is crucial for the development of efficient differential diagnostics methods to better distinguish between tumor entities even in small biopsies. Our results provide evidence that there are multiple molecular signatures which could help pathologists to diagnose small tissue samples with NSCLC. Novel findings include differentially expressed sets of secreted and non-secreted genes that may help in the diagnosis and classification of NSCLC on serum or tissue samples. The driver-gene search algorithm for integrating genomic data, mRNA and miRNA expression identified potential driver genes, which are useful for follow-up experimental validation. The findings of this study should help to instigate others to implement these in clinical practice.

### Data availability

The microarray data related to this paper have been submitted to the Array Express data repository at the European Bioinformatics Institute (http://www.ebi.ac.uk/arrayexpress/) under the accession numbers E-MTAB-1132 (GE), E-MTAB-1133 (CGH) and E-MTAB-1134 (MIR).

## Competing interests

The authors declare that they have no competing interests.

## Authors’ contributions

Conception and design: VL, JO, JCS, PD, JH and YP; Development of methodology: VL, CS, PD and YP; Acquisition of data (provided animals, acquired and managed patients, provided facilities, etc.): VL, JO, JH, JLu, PV, FD, AE, JC, BB, PG, FB, PV, JCS; Analysis and interpretation of data (e.g., statistical analysis, biostatistics, computational analysis): CS, CO, ZB, JG, BJ, GM, HR, SC, LL, PD, YP; writing, review, and/or revision of the manuscript: VL, CS, CO, JO, ZB, JLe, RN, MS, JCS, PD, YP; administrative, technical, or material support (i.e., reporting or organizing data, constructing databases): CO, JG, BJ, GM, HR, LL, SC; study supervision: VL, PD, JH, YP. All authors read and approved the final manuscript.

## Pre-publication history

The pre-publication history for this paper can be accessed here:

http://www.biomedcentral.com/1755-8794/6/53/prepub

## Supplementary Material

Additional file 1 **Figure S1.** Color map of the log2-expression ratio for AC and SCC patients using 657 gene-probes. Each column represents a patient and each row a probe. **Figure S2**: Boxplots of the log2-expression ratio for the top 5 secreted biomarkers found in Chemores and the corresponding log-expression values in Bild et al’s NSCLC data GSE3141. **Figure S3**: Boxplots of the log2-expression ratio for the top 5 non-secreted biomarkers and the corresponding log-expression values in Bild et al’s NSCLC data GSE3141. **Figure S4**: Drivers and number of their targeted genes given on the y-axis. **Figure S5**: P-values of Welch’s t test on –log10 scale (left) and fold change of driver genes’ expression (right) are given on x-axis; Number of targeted genes with a correlation coefficient at least 0.3 are given on the y-axis. Genes in the topright area are considered in a predictive model of lung cancer histology. **Table S1**: List of the 34 clusters of the most differential genomic regions between AC, LCC and SCC populations. **Table S2**: List of the 34 clusters of the most differential genomic regions between AC, LCC and SCC populations with the known genes within each cluster. **Table S3**: List of the 15 classifier-genes with the corresponding probes on Agilent 244K and Affymetrix U133 Plus 2.0 arrays. **Table S4**: List of the 10 potential biomarker genes with the corresponding probes on Agilent 244K and Affymetrix U133 Plus 2.0 arrays. **Table S5**: List of 4 candidate driver genes and 4 candidate driver miRNAs, their tumor expression levels and copy-number alteration status in AC and SCC. **Table S6**: Network enrichment analysis of target genes of MRPS22, NDRG1, RNF7 and hsa-miR-944. The top and bottom 20 ranked pathways are shown. Click here for file

## References

[B1] Jemal A Bray F Center MM Ferlay J Ward E Forman D Global cancer statistics CA Cancer J Clin 2011 61 69 90 10.3322/caac.20107 21296855

[B2] Malvezzi M Arfé A Bertuccio P Levi F La Vecchia C Negri E European cancer mortality predictions for the year 2011 Ann Oncol 2011 22 947 956 10.1093/annonc/mdq774 21303801

[B3] Bosetti C Levi F Lucchini F Negri E La Vecchia C Lung cancer mortality in European women: recent trends and perspectives Ann Oncol 2005 16 1597 1604 10.1093/annonc/mdi313 16014639

[B4] Spiro SG Silvestri GA One hundred years of lung cancer Am J Respir Crit Care Med 2005 172 523 529 10.1164/rccm.200504-531OE 15961694

[B5] Cagle PT Allen TC Dacic S Beasley MB Borczuk AC Chirieac LR Laucirica R Ro JY Kerr KM Revolution in lung cancer: new challenges for the surgical pathologist Arch Pathol Lab Med 2011 135 110 116 21204716 10.5858/2010-0567-RA.1

[B6] Tumor Analysis Best Practices Working Group Expression profiling–best practices for data generation and interpretation in clinical trials Nat Rev Genet 2004 5 229 237 14970825 10.1038/nrg1297

[B7] Olshen AB Venkatraman ES Lucito R Wigler M Circular binary segmentation for the analysis of array-based DNA copy number data Biostatistics 2004 5 4 557 572 10.1093/biostatistics/kxh008 15475419

[B8] Benjamini Y Hochberg Y Controlling the false discovery rate: a practical and powerful approach to multiple testing J Royal Statist Soc B 1995 57 289 300

[B9] Gentleman RC Carey VJ Bates DM Bolstad B Dettling M Dudoit S Ellis B Gautier L Ge Y Gentry J Hornik K Hothorn T Huber W Iacus S Irizarry R Leisch F Li C Maechler M Rossini AJ Sawitzki G Smith C Smyth G Tierney L Yang JY Zhang J Bioconductor: open software development for computational biology and bioinformatics Genome Biol 2004 5 R80 10.1186/gb-2004-5-10-r80 15461798 PMC545600

[B10] Smyth GK Linear models and empirical bayes methods for assessing differential expression in microarray experiments Stat Appl Genet Mol Biol 2004 3 1 3 10.2202/1544-6115.1027 16646809

[B11] Suo C Salim A Chia KS Pawitan Y Calza S Modified least-variant set normalization for miRNA microarray RNA 2010 16 12 2293 2303 10.1261/rna.2345710 20980676 PMC2995391

[B12] Akavia UD Litvin O Kim J Sanchez-Garcia F Kotliar D Causton HC Pochanard P Mozes E Garraway LA Pe’er D An integrated approach to uncover drivers of cancer Cell 2010 143 1005 1017 10.1016/j.cell.2010.11.013 21129771 PMC3013278

[B13] Teo SM Pawitan Y Kumar V Thalamuthu A Seielstad M Chia KS Salim A Multi-platform segmentation for joint detection of copy number variants Bioinformatics 2011 27 11 1555 1561 10.1093/bioinformatics/btr162 21471018

[B14] Teo SM Salim A Calza S Ku CS Chia KS Pawitan Y Identification of recurrent regions of copy-number variants across multiple individuals BMC Bioinforma 2010 11 147 10.1186/1471-2105-11-147 PMC2851607 20307285

[B15] Lee W Lee D Lee Y Pawitan Y Sparse canonical covariance analysis for high-throughput data Stat Appl Genet Mol Biol 2011 10 1 30

[B16] Alexeyenko A Lee W Pernemalm M Guegan J Dessen P Lazar V Lehtiö J Pawitan Y Network enrichment analysis: extension of gene-set enrichment analysis to gene networks BMC Bioinforma 2012 13 226 10.1186/1471-2105-13-226 PMC3505158 22966941

[B17] Griffiths-Jones S Grocock RJ Van Dongen S Bateman A Enright AJ miRBase: microRNA sequences, targets and gene nomenclature Nucleic Acids Res 2006 34 140 144 10.1093/nar/gkj430 16381832 PMC1347474

[B18] Bild AH Yao G Chang JT Wang Q Potti A Chasse D Joshi MB Harpole D Lancaster JM Berchuck A Olson JA Jr Marks JR Dressman HK West M Nevins JR Oncogenic pathway signatures in human cancers as a guide to targeted therapies Nature 2006 439 7074 353 357 10.1038/nature04296 16273092

[B19] Lu Y Govindan R Wang L Liu PY Goodgame B Wen W Sezhiyan A Pfeifer J Li YF Hua X Wang Y Yang P You M MicroRNA profiling and prediction of recurrence/relapse-free survival in stage I lung cancer Carcinogenesis 2012 0 1 9 doi:10.1093/carcin/bgs100 10.1093/carcin/bgs100 PMC3334512 22331473

[B20] Chitale D Gong Y Taylor BS Broderick S Brennan C Somwar R Golas B Wang L Motoi N Szoke J Reinersman JM Major J Sander C Seshan VE Zakowski MF Rusch V Pao W Gerald W Ladanyi M An integrated genomic analysis of lung cancer reveals loss of DUSP4 in EGFR-mutant tumors Oncogene 2009 28 31 2773 2783 doi: 10.1038/onc.2009.135. Epub 2009 Jun 15 10.1038/onc.2009.135 19525976 PMC2722688

[B21] Seo JS Ju YS Lee WC Shin JY Lee JK Bleazard T Lee J Jung YJ Kim JO Yu SB The transcriptional landscape and mutational profile of lung adenocarcinoma Genome Res 2012 22 11 2109 2119 doi:10.1101/gr.145144.112 10.1101/gr.145144.112 22975805 PMC3483540

[B22] Ju YS Lee WC Shin JY Lee S Bleazard T Won JK Kim YT Kim JI Kang JH Seo JS A transforming KIF5B and RET gene fusion in lung adenocarcinoma revealed from whole-genome and transcriptome sequencing Genome Res 2012 13 436 445 doi: 10.1101/gr.133645.111 22194472 10.1101/gr.133645.111 PMC3290779

[B23] Imielinski M Berger AH Hammerman PS Hernandez B Pugh TJ Hodis E Cho J Suh J Capelletti M Sivachenko A Sougnez C Auclair D Lawrence MS Stojanov P Cibulskis K Choi K De Waal L Sharifnia T Brooks A Greulich H Banerji S Zander T Seidel D Leenders F Ansén S Ludwig C Engel-Riedel W Stoelben E Wolf J Goparju C Mapping the hallmarks of lung adenocarcinoma with massively parallel sequencing Cell 2012 150 1107 1120 10.1016/j.cell.2012.08.029 22980975 PMC3557932

[B24] Cancer Genome Atlas Research Network Comprehensive genomic characterization of squamous cell lung cancers Nature 2012 489 519 525 10.1038/nature11404 22960745 PMC3466113

[B25] Borczuk AC Powell CA Expression profiling and lung cancer development Proc Am Thorac Soc 2007 4 127 132 10.1513/pats.200607-143JG 17202302

[B26] López-Malpartida AV Ludeña MD Varela G García PJ Differential ErbB receptor expression and intracellular signaling activity in lung adenocarcinomas and squamous cell carcinomas Lung Cancer 2009 65 25 33 10.1016/j.lungcan.2008.10.009 19046792

[B27] Da Cunha SG Saieg MA Geddie W Leighl N EGFR gene status in cytological samples of nonsmall cell lung carcinoma: controversies and opportunities Cancer Cytopathol 2011 119 80 91 10.1002/cncy.20150 21400669

[B28] Koo JS Kim SH EGFR and HER-2 status of non-small cell lung cancer brain metastasis and corresponding primary tumor Neoplasma 2011 58 27 34 10.4149/neo_2011_01_27 21067263

[B29] Gill RK Vazquez MF Kramer A Hames M Zhang L Heselmeyer-Haddad K Ried T Shilo K Henschke C Yankelevitz D Jen J The use of genetic markers to identify lung cancer in fine needle aspiration samples Clin Cancer Res 2008 14 7481 7487 10.1158/1078-0432.CCR-07-5242 19010865 PMC2586966

[B30] Donati V Boldrini L Dell’Omodarme M Prati MC Faviana P Camacci T Lucchi M Mussi A Santoro M Basolo F Fontanini G Osteopontin expression and prognostic significance in non-small cell lung cancer Clin Cancer Res 2005 11 6459 6465 10.1158/1078-0432.CCR-05-0541 16166420

[B31] Tang L Dai DL Su M Martinka M Li G Zhou Y Aberrant expression of collagen triple helix repeat containing 1 in human solid cancers Clin Cancer Res 2006 12 3716 3722 10.1158/1078-0432.CCR-06-0030 16778098

[B32] Mulvihill MS Kwon YW Lee S Fang LT Choi H Ray R Kang HC Mao JH Jablons D Kim IJ Gremlin is overexpressed in lung adenocarcinoma and increases cell growth and proliferation in normal lung cells PLoS One 2012 7 e42264 10.1371/journal.pone.0042264 22870311 PMC3411619

[B33] Kim M Yoon S Lee S Ha SA Kim HK Kim JW Chung J Gremlin-1 induces BMP-independent tumor cell proliferation, migration, and invasion PLoS One 2012 7 4 e35100 doi: 10.1371/journal.pone.0035100 10.1371/journal.pone.0035100 22514712 PMC3325980

[B34] Pecchio F Rapellino M Baldi S Casali V Libertucci D Coni F Tumor-associated trypsin inhibitor (TATI) in the diagnosis of lung cancer Scand J Clin Lab Invest Suppl 1991 207 63 64 1780695 10.3109/00365519109104630

[B35] Chen J Ye L Xie F Yang Y Zhang L Jiang WG Expression of bone morphogenetic protein 7 in lung cancer and its biological impact on lung cancer cells Anticancer Res 2010 30 1113 1120 20530416

[B36] Azuma K Kawahara A Hattori S Taira T Tsurutani J Watari K Shibata T Murakami Y Takamori S Ono M Izumi H Kage M Yanagawa T Nakagawa K Hoshino T Kuwano M NDRG1/Cap43/Drg-1 may predict tumor angiogenesis and poor outcome in patients with lung cancer J Thorac Oncol 2012 7 5 779 789 10.1097/JTO.0b013e31824c92b4 22481237

[B37] Sasaki H Yukiue H Kobayashi Y Moriyama S Nakashima Y Kaji M Fukai I Kiriyama M Yamakawa Y Fujii Y Expression of the sensitive to apoptosis gene, SAG, as a prognostic marker in nonsmall cell lung cancer Int J Cancer 2001 95 6 375 377 10.1002/1097-0215(20011120)95:6<375::AID-IJC1066>3.0.CO;2-L 11668520

