# Supplementary material for: Integrated molecular portrait of non-small cell lung cancers
Source: BMC Med Genomics. 2013 Dec 3;6:53. doi: 10.1186/1755-8794-6-53 (PMC4222074; doi:10.1186/1755-8794-6-53)
Supplement: Additional file 1 — Figure S1. Color map of the log2-expression ratio for AC and SCC patients using 657 gene-probes. Each column represents a patient and each row a probe. Figure S2: Boxplots of the log2-expression ratio for the top 5 secreted biomarkers found in Chemores and the corresponding log-expression values in Bild et al’s NSCLC data GSE3141. Figure S3: Boxplots of the log2-expression ratio for the top 5 non-secreted biomarkers and the corresponding log-expression values in Bild et al’s NSCLC data GSE3141. Figure S4: Drivers and number of their targeted genes given on the y-axis. Figure S5: P-values of Welch’s t test on –log10 scale (left) and fold change of driver genes’ expression (right) are given on x-axis; Number of targeted genes with a correlation coefficient at least 0.3 are given on the y-axis. Genes in the topright area are considered in a predictive model of lung cancer histology. Table S1: List of the 34 clusters of the most differential genomic regions between AC, LCC and SCC populations. Table S2: List of the 34 clusters of the most differential genomic regions between AC, LCC and SCC populations with the known genes within each cluster. Table S3: List of the 15 classifier-genes with the corresponding probes on Agilent 244K and Affymetrix U133 Plus 2.0 arrays. Table S4: List of the 10 potential biomarker genes with the corresponding probes on Agilent 244K and Affymetrix U133 Plus 2.0 arrays. Table S5: List of 4 candidate driver genes and 4 candidate driver miRNAs, their tumor expression levels and copy-number alteration status in AC and SCC. Table S6: Network enrichment analysis of target genes of MRPS22, NDRG1, RNF7 and hsa-miR-944. The top and bottom 20 ranked pathways are shown. [file 1755-8794-6-53-S1.docx]

**Supplementary Report**

**Lazar et al**., **Integrated molecular portrait of non-small cell lung cancers**

**Figure S1:** Color map of the log2-expression ratio for AC and SCC patients using 657 gene-probes. Each column represents a patient and each row a probe.


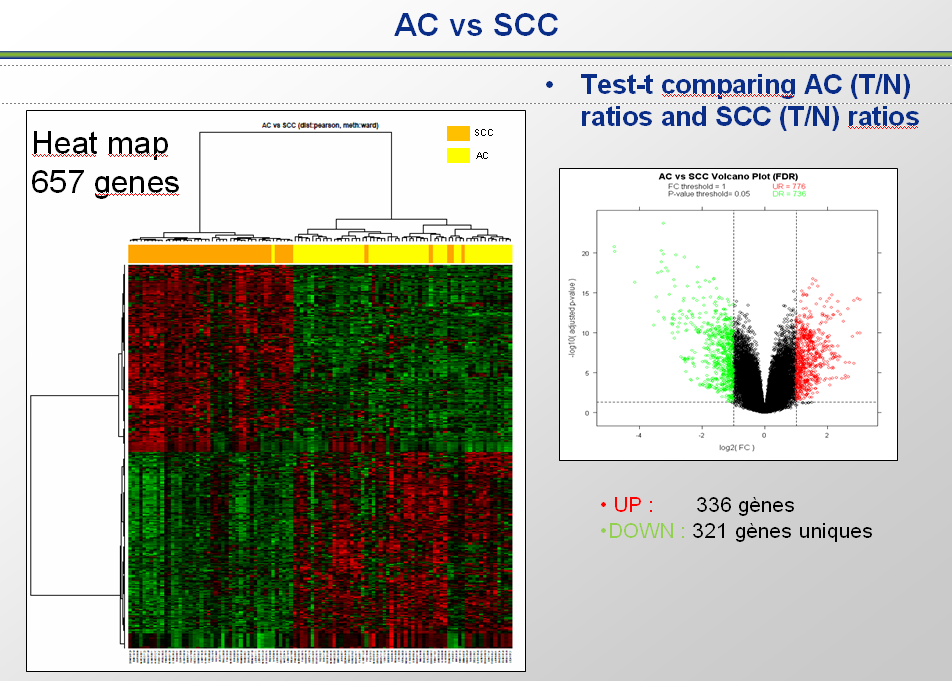


Figure S2: Boxplots of the log2-expression ratio for the top 5 secreted biomarkers found in Chemores and the corresponding log-expression values in Bild et al’s NSCLC data GSE3141.


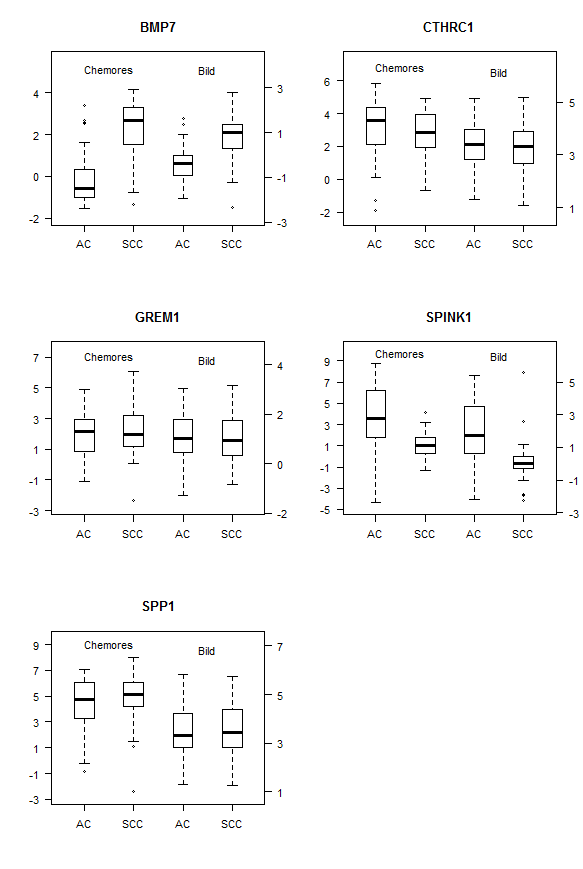
Figure S3: Boxplots of the log2-expression ratio for the top 5 non-secreted biomarkers and the corresponding log-expression values in Bild et al’s NSCLC data GSE3141.


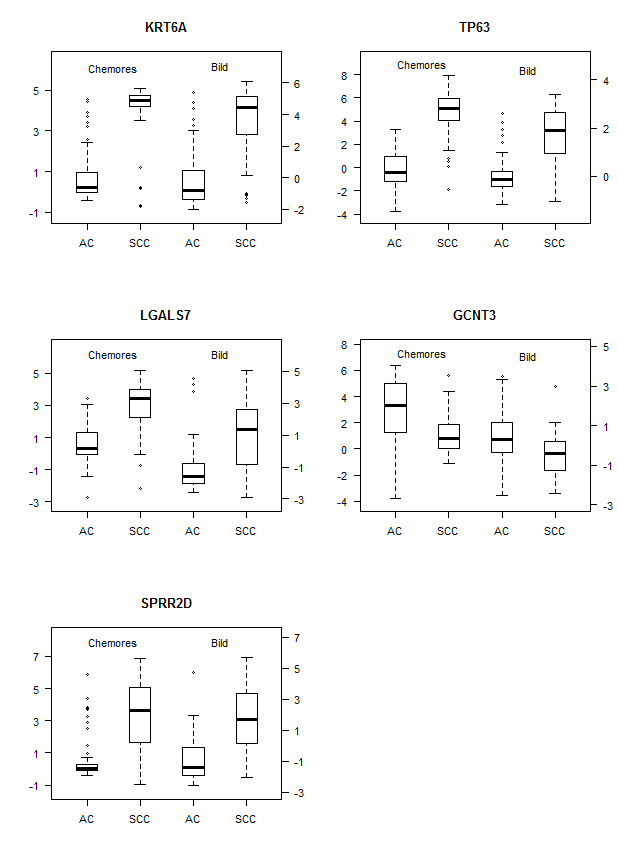


**Figure S4:** Drivers and number of their targeted genes given on the y-axis. When we count the targets reaching a certain correlation, the ranking of drivers based on the number of targets remains consistent across the correlation threshold.


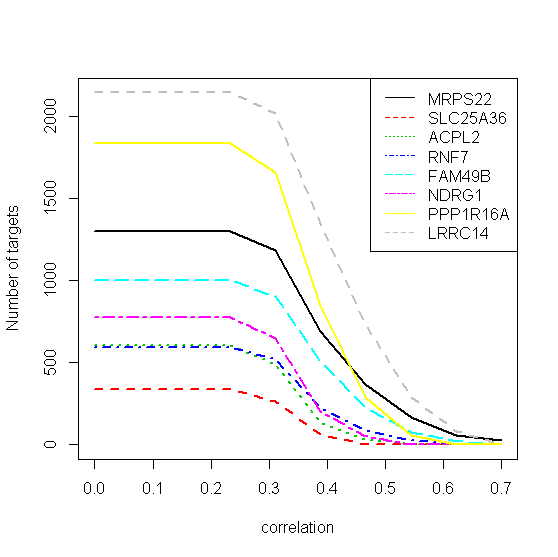


**Figure S5:** P-values of Welch’s t test on –log10 scale (left) and fold change of driver genes’ expression (right) are given on x-axis; Number of targeted genes with a correlation coefficient at least 0.3 are given on the y-axis. Genes in the topright area are considered in a predictive model of lung cancer histology.


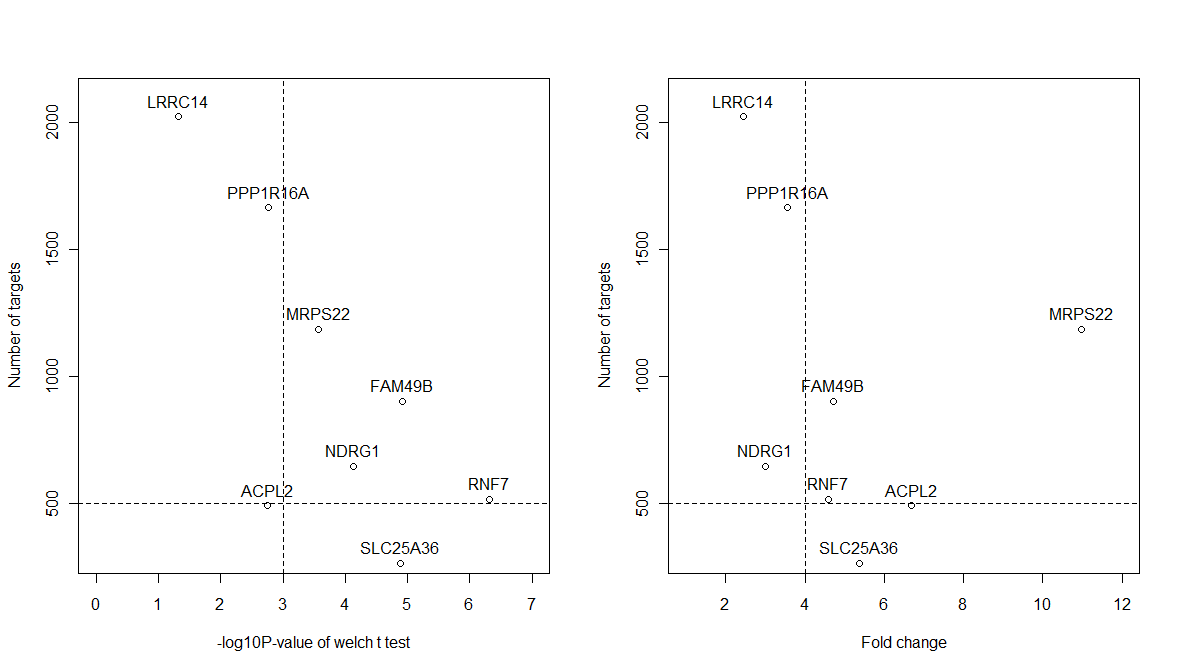


**Table S1:** List of the 34 clusters of the most differential genomic regions between AC, LCC and SCC populations.

| No. | Location | Width | Band | AC Gain | AC Loss | LCC Gain | LCC Loss | SCC Gain | SCC Loss | max AdjP.BH |
| --- | --- | --- | --- | --- | --- | --- | --- | --- | --- | --- |
| 1 | chr3:171313844-171381270 | 67 427 | 3q26.2 | 38% | 20% | 58% | 8% | 91% | 2% | 7,02E-06 |
| 2 | chr3:171388603-171634437 | 245 835 | 3q26.2 | 36% | 20% | 58% | 8% | 91% | 2% | 7,07E-06 |
| 3 | chr3:173208161-174220097 | 1 011 937 | 3q26.31 | 36% | 20% | 58% | 8% | 93% | 0% | 6,76E-06 |
| 4 | chr3:174237933-174305296 | 67 364 | 3q26.31 | 34% | 20% | 58% | 8% | 93% | 0% | 4,15E-06 |
| 5 | chr3:174558996-175734181 | 1 175 186 | 3q26.31 | 32% | 22% | 58% | 8% | 93% | 0% | 6,89E-06 |
| 6 | chr3:175745745-175860797 | 115 053 | 3q26.31 | 32% | 22% | 58% | 8% | 91% | 2% | 7,19E-06 |
| 7 | chr3:175895399-176230986 | 335 588 | 3q26.31 | 34% | 22% | 58% | 8% | 93% | 0% | 6,43E-06 |
| 8 | chr3:176241092-176504352 | 263 261 | 3q26.31 | 34% | 22% | 50% | 8% | 93% | 0% | 4,31E-06 |
| 9 | chr3:176510126-176635790 | 125 665 | 3q26.31 | 34% | 22% | 58% | 8% | 93% | 0% | 4,31E-06 |
| 10 | chr3:176667424-176951135 | 283 712 | 3q26.31 | 32% | 22% | 58% | 8% | 93% | 0% | 4,08E-06 |
| 11 | chr3:176963057-177314772 | 351 716 | 3q26.31 | 30% | 22% | 58% | 8% | 93% | 0% | 4,08E-06 |
| 12 | chr3:177345817-178056096 | 710 280 | 3q26.32 | 30% | 22% | 50% | 17% | 93% | 0% | 4,08E-06 |
| 13 | chr3:178136300-181504911 | 3 368 612 | 3q26.32 | 30% | 24% | 50% | 17% | 93% | 0% | 5,28E-06 |
| 14 | chr3:181533429-182211024 | 677 596 | 3q26.33 | 28% | 26% | 50% | 17% | 93% | 0% | 4,08E-06 |
| 15 | chr3:182247803-184754547 | 2 506 745 | 3q26.33 | 30% | 26% | 50% | 17% | 93% | 0% | 7,60E-06 |
| 16 | chr3:184761499-184982178 | 220 680 | 3q27.1 | 30% | 26% | 50% | 17% | 91% | 2% | 4,59E-06 |
| 17 | chr3:184993769-185026661 | 32 893 | 3q27.1 | 30% | 28% | 50% | 17% | 91% | 2% | 4,75E-06 |
| 18 | chr3:185038826-185775595 | 736 770 | 3q27.1 | 30% | 26% | 50% | 17% | 91% | 2% | 5,30E-06 |
| 19 | chr3:185782863-186268117 | 485 255 | 3q27.1 | 30% | 26% | 50% | 25% | 91% | 2% | 5,28E-06 |
| 20 | chr3:186282323-186967919 | 685 597 | 3q27.2 | 30% | 26% | 50% | 25% | 89% | 2% | 7,60E-06 |
| 21 | chr3:189722387-189858168 | 135 782 | 3q28 | 26% | 28% | 42% | 25% | 91% | 0% | 5,55E-06 |
| 22 | chr3:189868966-192382668 | 2 513 703 | 3q28 | 26% | 28% | 50% | 25% | 91% | 0% | 7,02E-06 |
| 23 | chr3:192931994-194614838 | 1 682 845 | 3q28 | 24% | 28% | 50% | 25% | 87% | 2% | 7,78E-06 |
| 24 | chr22:27355234-27570919 | 215 686 | 22q12.1 | 10% | 46% | 17% | 33% | 54% | 7% | 6,14E-06 |
| 25 | chr22:27582132-27707103 | 124 972 | 22q12.1 | 10% | 48% | 17% | 33% | 54% | 7% | 4,99E-06 |
| 26 | chr22:27716255-29342123 | 1 625 869 | 22q12.1 | 8% | 50% | 17% | 33% | 54% | 7% | 4,27E-06 |
| 27 | chr22:29347283-31299424 | 1 952 142 | 22q12.2 | 10% | 48% | 17% | 33% | 54% | 7% | 4,59E-06 |
| 28 | chr22:31314379-31565672 | 251 294 | 22q12.3 | 10% | 48% | 17% | 33% | 52% | 7% | 6,76E-06 |
| 29 | chr22:31666868-31862757 | 195 890 | 22q12.3 | 8% | 50% | 17% | 33% | 52% | 7% | 4,38E-06 |
| 30 | chr22:34415182-34504073 | 88 892 | 22q12.3 | 10% | 50% | 17% | 33% | 54% | 7% | 5,87E-06 |
| 31 | chr22:37852676-37856830 | 4 155 | 22q13.1 | 10% | 52% | 17% | 33% | 57% | 9% | 4,59E-06 |
| 32 | chr22:37861005-38341872 | 480 868 | 22q13.1 | 10% | 50% | 17% | 33% | 57% | 9% | 5,97E-06 |
| 33 | chr22:38354118-38577355 | 223 238 | 22q13.1 | 10% | 50% | 17% | 33% | 54% | 9% | 5,32E-06 |
| 34 | chr22:38735771-39069175 | 333 405 | 22q13.1 | 12% | 48% | 17% | 33% | 54% | 9% | 7,48E-06 |

**Table S2:** List of the 34 clusters of the most differential genomic regions between AC, LCC and SCC populations with the known genes within each cluster.

| ID | Band1 | COSMIC Oncogenes | COSMIC Tumor.Sup | COSMIC Ambiguous.Gene | Cancer.Census genes | Other genes of interest | miRNA |
| --- | --- | --- | --- | --- | --- | --- | --- |
| 1 | 3q26.2 | - | - | - | - | - | - |
| 2 | 3q26.2 | SKIL | - | PRKCI | - | CLDN11 | - |
| 3 | 3q26.31 | ECT2 | - | - | - | TNFSF10 | - |
| 4 | 3q26.31 | - | - | - | - | - | - |
| 5 | 3q26.31 | - | - | - | - | - | - |
| 6 | 3q26.31 | - | - | - | - | - | - |
| 7 | 3q26.31 | - | - | - | - | - | - |
| 8 | 3q26.31 | - | - | - | - | - | - |
| 9 | 3q26.31 | - | - | - | - | - | - |
| 10 | 3q26.31 | - | - | - | - | - | - |
| 11 | 3q26.31 | - | - | - | - | - | - |
| 12 | 3q26.32 | - | - | - | - | - | - |
| 13 | 3q26.32 | - | PIK3CA | - | - | TBL1XR1, ZMAT3 | - |
| 14 | 3q26.33 | - | - | - | - | - | - |
| 15 | 3q26.33 | DCUN1D1 | - | - | - | SOX2 | - |
| 16 | 3q27.1 | - | - | - | - | - | - |
| 17 | 3q27.1 | - | - | - | - | - | - |
| 18 | 3q27.1 | THPO | - | - | ECE2, POL2RH | ABCC5, DVL3, AP2M1, PSMD2 | hsa-miR-1224 |
| 19 | 3q27.1 | - | - | - | - | MAGEF1 | - |
| 20 | 3q27.2 | - | - | - | - | MAP3K13 | - |
| 21 | 3q28 | - | - | - | LPP | - | - |
| 22 | 3q28 | - | TP63 | - | LPP | TPRG1, CLDN1, CLDN16, IL1RAP | hsa-miR-28, hsa-miR-944 |
| 23 | 3q28 | - | - | - | - | FGF12, HRASLS | - |
| 24 | 22q12.1 | CHEK2 | - | - | - | - | - |
| 25 | 22q12.1 | - | - | - | - | - | - |
| 26 | 22q12.1 | EWSR1 | NF2 | - | NEFH, TCN2 | LIF, OSM | - |
| 27 | 22q12.2 | PATZ1 | YWHAH | - | TCN2, MORC2 | PLA2G3, PIK3IP1, SFI1 | - |
| 28 | 22q12.3 | - | - | - | - | - | - |
| 29 | 22q12.3 | - | - | - | - | - | - |
| 30 | 22q12.3 | - | - | - | - | - | - |
| 31 | 22q13.1 | - | - | - | - | - | - |
| 32 | 22q13.1 | PDGFB | - | - | MGAT3 | ATF4 | - |
| 33 | 22q13.1 | - | - | - | - | - | - |
| 34 | 22q13.1 | - | - | - | - | - | - |

**Table S3**: List of the 15 classifier-genes with the corresponding probes on Agilent 244K and Affymetrix U133 Plus 2.0 arrays.

| **Gene symbol** | **Agilent probe** | **Affy133 probe** |
| --- | --- | --- |
| S100A7 | A_23_P103310 | 205916_at |
| PKD2L1 | A_23_P12554 | 221061_at |
| TNNC2 | A_23_P131825 | 205388_at |
| CSTA | A_23_P170233 | 204971_at |
| FCGBP | A_23_P21495 | 203240_at |
|  |  |  |
| SLC1A7 | A_23_P325562 | 207355_at |
|  |  | 243623_at |
|  |  |  |
| TP63 | A_23_P327380 | 209863_s_at |
|  |  | 211194_s_at |
|  |  | 211195_s_at |
|  |  | 211834_s_at |
|  |  |  |
| WDR66 | A_23_P363275 | 230193_at |
|  |  |  |
| TFPI2 | A_23_P393620 | 209277_at |
|  |  | 209278_s_at |
|  |  |  |
| NR0B1 | A_23_P73632 | 206644_at |
|  |  | 206645_s_at |
|  |  |  |
| TESC | A_23_P76538 | 218872_at |
|  |  |  |
| APOC1 | A_24_P109214 | 204416_x_at |
|  |  | 213553_x_at |
|  |  |  |
| AKR1C1 | A_24_P220947 | 204151_x_at |
|  |  | 209699_x_at |
|  |  | 211653_x_at |
|  |  | 216594_x_at |
|  |  | 204151_x_at |
|  |  | 209699_x_at |
|  |  | 211653_x_at |
|  |  | 216594_x_at |
|  |  |  |
| XAGE1 | A_24_P271696 |  |
|  |  |  |
|  |  | 220057_at |
|  |  |  |
|  |  |  |
|  |  |  |
| SPTB | A_32_P134968 | 208416_s_at |
|  |  | 214145_s_at |

**Table S4**: List of the 10 potential biomarker genes with the corresponding probes on Agilent 244K and Affymetrix U133 Plus 2.0 arrays.

| **Gene symbol** | **Agilent probe** | **Affy133 probe** |
| --- | --- | --- |
| BMP7 | A_23_P68487 | 209591_s_at |
|  |  | 211259_s_at |
|  |  | 209590_at |
|  |  |  |
| CTHRC1 | A_23_P111888 | 225681_at |
|  |  |  |
| GREM1 | A_23_P432947 | 218468_s_at |
|  |  | 218469_at |
|  |  |  |
| SPINK1 | A_23_P214079 | 206239_s_at |
| SPP1 | A_23_P7313 | 209875_s_at |
|  |  |  |
| KRT6A | A_23_P87653 | 209125_at |
|  |  | 209126_x_at |
|  |  | 214580_x_at |
|  |  |  |
| TP63 | A_23_P327380 | 209863_s_at |
|  |  | 211194_s_at |
|  |  | 211195_s_at |
|  |  | 211834_s_at |
|  |  |  |
| LGALS7 | A_24_P348118 | 206400_at |
| GCNT3 | A_23_P151915 | 219508_at |
| SPRR2D | A_23_P11644 | 208539_x_at |

**Table S5:** List of 4 candidate driver genes and 4 candidate driver miRNAs, their tumor expression levels and copy-number alteration status in AC and SCC.

| Driver genes/miRNAs | chr | start | end | Overexpression in  T vs N | | Individuals with amplifications and deletions | |
| --- | --- | --- | --- | --- | --- | --- | --- |
|  |  |  |  | AC (%) | SCC (%) | AC(%)  amp/del | SCC(%) amp/del |
| MRPS22 | 3 | 140551801 | 140558467 | 30 | 78 | 2 / 2 | 16 / 0 |
| RNF7 | 3 | 142942186 | 142944941 | 50 | 88 | 5 / 0 | 22 / 0 |
| NDRG1 | 8 | 134319466 | 134377041 | 62 | 86 | 7 / 0 | 14 / 0 |
| FAM49B | 8 | 130853716 | 131029375 | 70 | 74 | 13 / 0 | 14 / 0 |
|  |  |  |  |  |  |  |  |
| hsa-miR-16-2* | 3 | 160122586 | 160122606 | 36 | 40 | 5 / 2 | 28 / 0 |
| hsa-miR-570 | 3 | 195426334 | 195426352 | 52 | 62 | 4 / 2 | 36 / 0 |
| hsa-miR-944 | 3 | 189547767 | 189547785 | 59 | 90 | 5 / 2 | 40 / 0 |
| hsa-miR-31* | 9 | 21512160 | 21512178 | 70 | 68 | 0 / 11 | 2 / 22 |

**Table S6:** Network enrichment analysis of target genes of MRPS22, NDRG1, RNF7 and hsa-miR-944. The top and bottom 20 ranked pathways are shown. The z-scores can be used as a measure of activation of a pathway, with positive values indicating activated pathway and negative values indicating the opposite.

**MRPS22**

| **PATH ID** | **PATH NAME** | **Number links** | | **Expected links** | | **Number of Genes in pathway** | | **Z-score** | | **FDR** | |
| --- | --- | --- | --- | --- | --- | --- | --- | --- | --- | --- | --- |
| hsa03030 | DNA replication | | 458 | | 269 | | 36 | | 8.53 | | 0.027 |
| hsa04110 | Cell cycle | | 822 | | 533 | | 125 | | 8.18 | | 0.027 |
| hsa03013 | RNA transport | | 1150 | | 863 | | 152 | | 8.03 | | 0.027 |
| hsa03430 | Mismatch repair | | 271 | | 160 | | 23 | | 7.63 | | 0.027 |
| hsa03440 | Homologous recombination | | 196 | | 113 | | 28 | | 6.99 | | 0.027 |
| hsa03420 | Nucleotide excision repair | | 362 | | 229 | | 45 | | 6.88 | | 0.027 |
| hsa03410 | Base excision repair | | 225 | | 138 | | 34 | | 6.68 | | 0.027 |
| hsa03050 | Proteasome | | 502 | | 362 | | 45 | | 6.40 | | 0.027 |
| hsa03015 | mRNA surveillance pathway | | 473 | | 368 | | 83 | | 5.14 | | 0.027 |
| hsa03040 | Spliceosome | | 1099 | | 956 | | 128 | | 5.09 | | 0.027 |
| hsa04114 | Oocyte meiosis | | 549 | | 440 | | 113 | | 4.98 | | 0.027 |
| hsa04120 | Ubiquitin mediated proteolysis | | 523 | | 431 | | 136 | | 4.81 | | 0.027 |
| hsa04115 | p53 signaling pathway | | 177 | | 121 | | 69 | | 4.63 | | 0.027 |
| hsa03018 | RNA degradation | | 436 | | 354 | | 71 | | 4.46 | | 0.027 |
| hsa04914 | Progesterone-mediated oocyte maturation | | 353 | | 288 | | 87 | | 4.07 | | 0.027 |
| hsa00240 | Pyrimidine metabolism | | 485 | | 420 | | 99 | | 3.47 | | 0.027 |
| hsa03450 | Non-homologous end-joining | | 89 | | 66 | | 14 | | 3.44 | | 0.027 |
| hsa00601 | Glycosphingolipid biosynthesis - lacto and neolacto series | | 26 | | 15 | | 26 | | 3.06 | | 0.027 |
| hsa03008 | Ribosome biogenesis in eukaryotes | | 446 | | 398 | | 81 | | 2.71 | | 0.027 |
| hsa05012 | Parkinson's disease | | 475 | | 427 | | 130 | | 2.63 | | 0.027 |
|  |  | |  | |  | |  | |  | |  |
|  |  | |  | |  | |  | |  | |  |
|  |  | |  | |  | |  | |  | |  |
|  |  | |  | |  | |  | |  | |  |
|  |  | |  | |  | |  | |  | |  |
|  |  | |  | |  | |  | |  | |  |
|  |  | |  | |  | |  | |  | |  |
| **NDRG1** |  | |  | |  | |  | |  | |  |
| **PATH ID** | **PATH NAME** | | **Number links** | | **Expected links** | | **Number of Genes in pathway** | | **Z-score** | | **FDR** |
| hsa04810 | Regulation of actin cytoskeleton | | 463 | | 287 | | 214 | | 7.60 | | 0.029 |
| hsa05100 | Bacterial invasion of epithelial cells | | 245 | | 137 | | 71 | | 7.55 | | 0.029 |
| hsa04144 | Endocytosis | | 421 | | 279 | | 202 | | 7.45 | | 0.029 |
| hsa05131 | Shigellosis | | 218 | | 133 | | 62 | | 7.24 | | 0.029 |
| hsa04666 | Fc gamma R-mediated phagocytosis | | 299 | | 168 | | 95 | | 7.21 | | 0.029 |
| hsa04722 | Neurotrophin signaling pathway | | 329 | | 206 | | 127 | | 7.10 | | 0.029 |
| hsa04662 | B cell receptor signaling pathway | | 190 | | 109 | | 75 | | 6.69 | | 0.029 |
| hsa04660 | T cell receptor signaling pathway | | 245 | | 155 | | 108 | | 6.63 | | 0.029 |
| hsa04664 | Fc epsilon RI signaling pathway | | 205 | | 115 | | 79 | | 6.62 | | 0.029 |
| hsa04062 | Chemokine signaling pathway | | 332 | | 221 | | 189 | | 6.46 | | 0.029 |
| hsa04142 | Lysosome | | 224 | | 137 | | 121 | | 6.29 | | 0.029 |
| hsa04145 | Phagosome | | 302 | | 207 | | 154 | | 6.25 | | 0.029 |
| hsa04670 | Leukocyte transendothelial migration | | 218 | | 132 | | 117 | | 6.25 | | 0.029 |
| hsa05120 | Epithelial cell signaling in Helicobacter pylori infection | | 176 | | 114 | | 68 | | 6.23 | | 0.029 |
| hsa04510 | Focal adhesion | | 353 | | 251 | | 200 | | 6.00 | | 0.029 |
| hsa04380 | Osteoclast differentiation | | 183 | | 125 | | 128 | | 5.95 | | 0.029 |
| hsa05145 | Toxoplasmosis | | 237 | | 165 | | 133 | | 5.94 | | 0.029 |
| hsa04370 | VEGF signaling pathway | | 172 | | 103 | | 76 | | 5.86 | | 0.029 |
| hsa04650 | Natural killer cell mediated cytotoxicity | | 203 | | 130 | | 136 | | 5.61 | | 0.029 |
| hsa04012 | ErbB signaling pathway | | 213 | | 145 | | 87 | | 5.49 | | 0.029 |
|  |  | |  | |  | |  | |  | |  |
|  |  | |  | |  | |  | |  | |  |
|  |  | |  | |  | |  | |  | |  |
|  |  | |  | |  | |  | |  | |  |
|  |  | |  | |  | |  | |  | |  |
|  |  | |  | |  | |  | |  | |  |
|  |  | |  | |  | |  | |  | |  |
|  |  | |  | |  | |  | |  | |  |
|  |  | |  | |  | |  | |  | |  |
|  |  | |  | |  | |  | |  | |  |
|  |  | |  | |  | |  | |  | |  |
|  |  | |  | |  | |  | |  | |  |
| **RNF7** |  | |  | |  | |  | |  | |  |
| **PATH ID** | **PATH NAME** | | **Number links** | | **Expected links** | | **Number of Genes in pathway** | | **Z-score** | | **FDR** |
| hsa05412 | Arrhythmogenic right ventricular cardiomyopathy (ARVC) | | 201 | | 37 | | 74 | | 9.46 | | 0.024 |
| hsa04510 | Focal adhesion | | 471 | | 155 | | 200 | | 9.38 | | 0.024 |
| hsa04512 | ECM-receptor interaction | | 154 | | 24 | | 85 | | 9.36 | | 0.024 |
| hsa05200 | Pathways in cancer | | 548 | | 216 | | 327 | | 9.17 | | 0.024 |
| hsa04810 | Regulation of actin cytoskeleton | | 442 | | 181 | | 214 | | 9.17 | | 0.024 |
| hsa05146 | Amoebiasis | | 192 | | 54 | | 106 | | 9.11 | | 0.024 |
| hsa04520 | Adherens junction | | 215 | | 76 | | 73 | | 8.81 | | 0.024 |
| hsa05145 | Toxoplasmosis | | 264 | | 100 | | 133 | | 8.60 | | 0.024 |
| hsa04670 | Leukocyte transendothelial migration | | 223 | | 79 | | 117 | | 8.60 | | 0.024 |
| hsa04610 | Complement and coagulation cascades | | 65 | | 11 | | 69 | | 8.52 | | 0.024 |
| hsa05410 | Hypertrophic cardiomyopathy (HCM) | | 128 | | 38 | | 83 | | 8.48 | | 0.024 |
| hsa04010 | MAPK signaling pathway | | 360 | | 176 | | 268 | | 8.36 | | 0.024 |
| hsa05100 | Bacterial invasion of epithelial cells | | 228 | | 88 | | 71 | | 8.36 | | 0.024 |
| hsa05222 | Small cell lung cancer | | 167 | | 55 | | 85 | | 8.34 | | 0.024 |
| hsa05218 | Melanoma | | 136 | | 44 | | 71 | | 8.32 | | 0.024 |
| hsa05414 | Dilated cardiomyopathy | | 121 | | 42 | | 90 | | 8.30 | | 0.024 |
| hsa04012 | ErbB signaling pathway | | 221 | | 86 | | 87 | | 8.18 | | 0.024 |
| hsa00590 | Arachidonic acid metabolism | | 79 | | 20 | | 59 | | 8.16 | | 0.024 |
| hsa04640 | Hematopoietic cell lineage | | 78 | | 19 | | 88 | | 8.11 | | 0.024 |
| hsa04912 | GnRH signaling pathway | | 195 | | 83 | | 101 | | 8.10 | | 0.024 |

| **hsa-miR-944** | |  |  |  |  |  |
| --- | --- | --- | --- | --- | --- | --- |
| **PATH ID** | **PATH NAME** | **Number links** | **Expected links** | **Number of Genes in pathway** | **Z-score** | **FDR** |
| hsa00982 | Drug metabolism - cytochrome P450 | 452 | 312 | 73 | 6.51 | 0.040 |
| hsa00980 | Metabolism of xenobiotics by cytochrome P450 | 464 | 321 | 71 | 6.40 | 0.040 |
| hsa03030 | DNA replication | 1201 | 991 | 36 | 6.39 | 0.040 |
| hsa00830 | Retinol metabolism | 372 | 259 | 64 | 5.98 | 0.040 |
| hsa03430 | Mismatch repair | 729 | 593 | 23 | 5.95 | 0.040 |
| hsa03420 | Nucleotide excision repair | 1015 | 840 | 45 | 5.88 | 0.040 |
| hsa03440 | Homologous recombination | 530 | 422 | 28 | 5.76 | 0.040 |
| hsa04110 | Cell cycle | 2199 | 1938 | 125 | 5.72 | 0.040 |
| hsa00591 | Linoleic acid metabolism | 195 | 134 | 30 | 5.07 | 0.040 |
| hsa03013 | RNA transport | 3450 | 3175 | 152 | 5.01 | 0.040 |
| hsa00590 | Arachidonic acid metabolism | 280 | 208 | 59 | 4.94 | 0.040 |
| hsa00053 | Ascorbate and aldarate metabolism | 171 | 120 | 26 | 4.60 | 0.040 |
| hsa00360 | Phenylalanine metabolism | 103 | 69 | 17 | 4.35 | 0.040 |
| hsa03018 | RNA degradation | 1451 | 1298 | 71 | 4.32 | 0.040 |
| hsa00340 | Histidine metabolism | 168 | 124 | 29 | 4.02 | 0.040 |
| hsa00350 | Tyrosine metabolism | 221 | 174 | 41 | 4.01 | 0.040 |
| hsa03040 | Spliceosome | 3730 | 3514 | 128 | 3.96 | 0.040 |
| hsa00040 | Pentose and glucuronate interconversions | 250 | 198 | 32 | 3.88 | 0.040 |
| hsa03060 | Protein export | 477 | 405 | 23 | 3.67 | 0.040 |
| hsa00380 | Tryptophan metabolism | 246 | 196 | 42 | 3.35 | 0.040 |
|  |  |  |  |  |  |  |
|  |  |  |  |  |  |  |
|  |  |  |  |  |  |  |
|  |  |  |  |  |  |  |
|  |  |  |  |  |  |  |
|  |  |  |  |  |  |  |
|  |  |  |  |  |  |  |
